# Detection of Gaseous Plumes using Basis Vectors

**DOI:** 10.3390/s90503205

**Published:** 2009-04-27

**Authors:** Lawrence Chilton, Stephen Walsh

**Affiliations:** PO Box 999, Pacific Northwest National Laboratory, Richland, WA 99352 E-Mail: stephen.walsh@pnl.gov

**Keywords:** Plumes, detection, LWIR, basis vectors, generalized least squares

## Abstract

Detecting and identifying weak gaseous plumes using thermal imaging data is complicated by many factors. There are several methods currently being used to detect plumes. They can be grouped into two categories: those that use a chemical spectral library and those that don't. The approaches that use chemical libraries include physics-based least squares methods (matched filter). They are “optimal” only if the plume chemical is actually in the search library but risk missing chemicals not in the library. The methods that don't use a chemical spectral library are based on a statistical or data analytical transformation applied to the data. These include principle components, independent components, entropy, Fourier transform, and others. These methods do not explicitly take advantage of the physics of the signal formulation process and therefore don't exploit all available information in the data. This paper describes generalized least squares detection using gas spectra, presents a new detection method using basis vectors, and compares detection images resulting from applying both methods to synthetic hyperspectral data.

## 1. Introduction

Detecting and identifying weak gaseous plumes using infrared measurement instruments is a challenge that receives continual attention. Burr and Hengartner [1] have provided a comprehensive review of this problem. There are several methods currently being used to detect plumes. They can be grouped into two categories, those that use chemical libraries and those that don't.

The approaches that use chemical libraries include least squares approaches (i.e., the use of matched filters) and other physics-based methods [1–3]. These methods search for evidence of a specific chemical signature in an image by using a library of laboratory measured chemical spectra. A “detection image” is created for each chemical by applying some variation of a matched filter to each pixel in the image [1]. An analyst then looks for “plume-like” shapes in each detection image. Shortcomings of these methods include the following. They are “optimal” only if the plume chemical is actually in the search library but risk missing chemicals not in the library. Also, due to the uncertainties in the environmental parameters, they risk not detecting plumes that are distorted by atmospheric and other environmental uncertainties. These approaches have the ability to simultaneously detect the plume and potentially identify the plume's chemical constituents, which is a key difference between them and the methods we describe next which only detect plumes.

Methods that don't use a chemical spectral library are based on a statistical or data analytical transformation applied to the data. These include principle components, independent components, entropy, Fourier transform, and several other combinations or modifications, e.g. see [4]. These methods do not explicitly take advantage of the signal formulation physics, and therefore don't exploit all available information in the data. They also risk producing features/artifacts that have no obvious physics-based interpretation. Finally, they also rely on an analyst to recognize “plume-like” objects and distinguish them from non-plume features.

In this paper we introduce a plume detection method that avoids some short-comings of both previously mentioned methods but also has features in common with both. This new method is not intended to replace current methods but rather to complement them. It is physics-based but it is not defined by the members of any specific collection of chemicals, large or small. Instead it uses surrogate chemical spectra which form a basis set for the set of all possible chemical spectra. The method has been applied to both real and synthetic hyperspectral imagery. Only the results from synthetic data are presented here but results on real datacubes are similar. Section 2 presents the physics-based model. Section 3 presents matched filter detection and the basis vector method. Section 4 presents experimental results on a synthetic HSI datacube and conclusions are presented in Section 5.

## 2. Physics-based Radiance Model

In this section we present the three-layer physics-based radiance model which describes the basic physics of radiative transfer in the context of plume detection [1, 3, 5]. We present the model as a function of wavelength, *λ* (in *μ*m).

This model can be written as:

(1) $$L_{\text{obs}}(\lambda )=\tau_{a}(\lambda )[(1‐\tau_{p}(\lambda ))B(T_{p};\lambda )+\tau_{p}(\lambda )L_{g}(\lambda )]+L_{u}(\lambda )+n(\lambda )$$

where *L_obs_*(*λ*) represents sensor-recorded radiance in *W*/(*m*^2^ * *sr* * *μ*m) at wavelength *λ* (*μm*), *τ_a_*(*λ*) and *τ_p_*(*λ*) are dimensionless terms representing the atmosphere and plume transmissivity, respectively, *B*(*T_p_*;*λ*) has radiance units and is Planck's Blackbody function at wavelength *λ* and plume temperature *T_p_* (K), *L_g_*(*λ*) and *L_u_*(*λ*) are the ground-leaving and atmospheric upwelling radiances, respectively, and *n*(*λ*) includes unmodeled effects and sensor noise [6].

Following the convention of [1, 7], we model the ground-leaving radiance as:

(2) $$L_{g}(\lambda )={ɛ}_{g}(\lambda )B(T_{p};\lambda )$$

where *ε_g_*(*λ*) is a dimensionless quantity representing the emissivity of the ground at wavelength *λ*, with 0 ≤ *ε_g_*(*λ*) ≤ 1, and *T_g_* represents the ground temperature. Note that this formulation ignores the reflected atmospheric downwelling radiance. This assumption is reasonable in the Longwave Infrared band (LWIR) because the reflected radiance contribution to observed signal is negligible [7].

The Beer-Bourger-Lambert Law [8] gives an explicit expression for the transmissivity of a plume in terms of the chemical effluent's concentration path-length, *c* (with *c* measured in parts-per-million-meter, denoted *ppm-m*), as follows:

(3) $$\tau_{p}=\text{exp}\left(‐\sum_{j=1}^{N_{c}}A_{j}(\lambda )c_{j}\right)$$

where *A_j_*(*λ*) is the absorbance coefficient of chemical *j* in (*ppm-m*)^−1^ [8] and *N_c_* denotes the number of chemicals in the plume. For optically thin plumes, this term is well approximated by the first two terms in a Taylor Series expansion [1]. This gives:

(4) $$\tau_{p}(\lambda )≅1‐\sum_{j=1}^{N_{c}}A_{j}(\lambda )c_{j}$$

for small *c*.

Substituting Equation (4) into Equation (1) yields the working gas-plume linearized model:

(5) $$L_{\text{obs}}(\lambda )=\tau_{a}(\lambda )[B(T_{p};\lambda )‐L_{g}(\lambda )]\sum_{j=1}^{N_{c}}A_{j}(\lambda )c_{j}+\tau_{a}(\lambda )L_{g}(\lambda )+L_{u}(\lambda )n(\lambda )$$

where the noise term *n*(*λ*) now includes the approximation error due to application of Equation (4). Equation (5) shows that the sensor incident radiance *L_obs_*(*λ*) can be represented as an additive layering of the chemical signal $\tau_{a}(\lambda )[B(T_{p};\lambda )‐L_{g}(\lambda )]\sum_{j=1}^{N_{c}}A_{j}(\lambda )c_{j}$, ground radiance transmitted through the atmosphere, *τ_a_*(*λ*)*L_g_*(*λ*), atmospheric upwelling radiance, *L_u_*(*λ*), and noise, *n*(*λ*). This formulation motivates *scene whitening* i.e. background radiance subtraction and decorrelation. In the next section we discuss scene whitening in the context of the detection methods.

## 3. Detection Method Formulations

In this section we discuss the matched filter or generalized least squares (GLS) approach to gas detection and the Basis Vector Detection (BVD) method.

### 3.1. Matched Filter

As a hyperspectral sensing instrument records radiance at a number of channels, we will present the physics-based model and data processing in vector-matrix notation. We will also restrict our exploration to the single chemical case.

Let *N_λ_* denote the number of spectral channels recorded by the instrument. The physics-based model in Equation (5) can be written in vector form as:

(6) $${\text{L}}_{\text{obs}}=\tau_{a}⊙(\text{B}(T_{p})‐{\text{L}}_{g})⊙\text{A}c+\tau_{a}⊙{\text{L}}_{g}+{\text{L}}_{u}+\text{n}$$

where **bold** terms represent *N_λ_* × 1 vectors and ⊙ denotes the Hadamard product (element-wise multiplication).

An initial step is to remove the background radiance. To do this we compute the scene-wide mean radiance ***L̅*** = ***τ****_a_* ⊙ ***L̅****_g_* + ***L****_u_* +***n̅*** while assuming constant atmospheric terms ***τ****_a_* and ***L****_u_*. This provides a reasonable approximation to the background (non-plume) radiance provided the plume(s) are small (up to a few tens of pixels out of tens of thousands) and weak (in concentration and temperature contrast with the background) [1]. We subtract ***L̅*** from both sides of Equation (6) to arrive at:

(7) $$\begin{array}{ll} \text{r} & ={\text{L}}_{\text{obs}}‐\bar{\text{L}}\\ & =\tau_{a}⊙(\text{B}(T_{p})‐{\text{L}}_{g})⊙\text{A}c+z \end{array}$$

where ***r*** is the mean subtracted pixel radiance and we assume ***z*** = ***τ****_a_* ⊙ (***L****_g_* − ***L̅****_g_*) + (***n−n̅***) is zero mean with covariance matrix Σ. Under the weak gaseous plume assumption, the scene-wide covariance Σˆ will provide a reasonable approximation to Σ [1].

Equation (7) motivates the GLS approach to gas detection. However, the most appropriate treatment requires knowledge of the nuisance parameters ***τ****_a_*, ***T****_p_* and ***L****_g_*. The atmospheric transmissivity ***τ****_a_* can be estimated using an in scene method such as ISAC [9] or can be modeled using radiosonde data and estimated by MODTRAN [10]. There are several methods that can provide in scene estimates of ***L****_g_* or attempt to retrieve surface emissivity and temperature [5, 11].

For this paper we take the worst case approach which is to assume no information about the nuisance parameters is available. If such information is available, it can be incorporated to improve performance. We consider finding the GLS solution *βˆ* to:

(8) r=Aβ+z

and consider large values of *βˆ* as evidence of chemical signature due to ***A*** in the pixel [1]. Explicitly we will compute:

(9) $$\hat{\beta }={\left(\text{A}\prime {\hat{\sum }}^{‐1}\text{A}\right)}^{‐1}\text{A}\prime {\hat{\sum }}^{‐1}r.$$

In summary, for each chemical in the library of candidates, a detection image is constructed from the results of computing *βˆ* on each pixel. The “ *βˆ*-image” is then inspected for groups of contiguous pixels with large *βˆ* values, perhaps showing a “plume-like” shape.

The success of this method depends on having the plume chemicals in the search library. If the plume chemicals are not known, choosing the chemical search library can be a challenge. Due to these factors, we developed a plume detection method independent of the chemicals in the plume but still using the physics-based model, which we present in the next subsection.

### 3.2. Basis Vector Detection (BVD)

BVD is based on a fixed set of surrogate chemical spectra that are defined by the resolution of the imaging instrument and not by the chemical library. We choose the set of surrogate spectra to span the vector space ℝ*^Nλ^*, the set of all possible spectra produced by a hyperspectral instrument with *N_λ_* channels. There are several ways to construct a basis for this space. For this work we explore the coordinate unit vectors, which is the simplest basis in the sense that it is both orthogonal and sparse. We intend to explore the application of other basis sets in future work.

Suppose the hyperspectral instrument collects energy at a set of wavelengths ***λ*** = {*λ*_1_, *λ*_2_,…,*λ_Nλ_*} in the LWIR. Then the surrogate set of spectra is **E** = {***e***_1_,***e***_2_,…,***e****_Nλ_*}, where***e****_k_* = (0,…,0,1,0,…,0)′ has dimension *N_λ_* with the 1 in position *k*. Each basis vector is used to search for large deviations in its respective direction and these may be associated with the effects of a chemical effluent. One immediate advantage of this approach is that the size of the surrogate library E is fixed and can be used to detect every chemical or combination of chemicals in any chemical library, no matter how large.

The goal of BVD is to produce a detection image for each channel *k* which will be inspected for “plume-like” shapes. To do this we will consider how well the basis vector *e_k_* fits the whitened pixel ***r*** = ***L****_obs_ −* ***L̅*** by finding the GLS solution to:

(10) $$\text{r}={\text{e}}_{k}\beta_{k}+\text{z}.$$

Thus we compute:

(11) $${\hat{\beta }}_{k}={\left(e_{k}^{\prime }{\hat{\sum }}^{‐1}e_{k}\right)}^{‐1}e_{k}^{\prime }{\hat{\sum }}^{‐1}r$$

for each channel *k*. We consider large values of *βˆ_k_* as evidence of the effects of a chemical effluent. This analysis is applied to every pixel producing a “ *βˆ_k_*-image” for each channel *λ_k_*. This set of detection images is scanned by an analyst to find “plume-like” shapes.

### 3.3. BVD Justification

In this section we analyze the relationship between *βˆ_k_* in Equation (11) and the chemical absorbance spectra. With some simplifying assumptions, we show that, when the plume is composed of one chemical, *βˆ_k_* is proportional to the magnitude of the chemical absorbance in channel *k*. In experimentation we observe that the detection images for channels in which a chemical exhibits large absorbance features sometimes contain strong evidence for plume detection, i.e. we observe large *βˆ_k_* when chemical ***A*** has a large absorbance value *A_k_* in channel *k*. We show that *βˆ_k_* is proportional to *A_k_* if we ignore sensor noise. This simplification is reasonable in this case because our goal is to isolate the relationship between *βˆ_k_* and *A_k_*.

We recall our assumption of only one chemical in the plume and since ***L****_g_* = *ε_g_* ⊙ ***B***(***T****_g_*), we substitute this into Equation (7) to get:

(12) $$\text{r}=\tau_{a}⊙(\text{B}(T_{p})‐{ɛ}_{g}⊙\text{B}(T_{g}))⊙\text{A}c+z.$$

We substitute Equation (12) into Equation (11) and set *c* = 1 to get:

(13) $$\begin{array}{ll} {\hat{\beta }}_{k} & ={\left(e_{k}^{\prime }{\hat{\sum }}^{‐1}e_{k}\right)}^{‐1}e_{k}^{\prime }{\hat{\sum }}^{‐1}r\\ & ≅\frac{1}{{\hat{\sum }}_{kk}^{‐1}}e_{k}^{\prime }{\hat{\sum }}^{‐1}\tau_{a}⊙(\text{B}(T_{p})‐{ɛ}_{g}⊙\text{B}(T_{g}))\text{A}. \end{array}$$

Ignoring sensor noise it is possible to express the covariance matrix Σˆ as a function of the nuisance parameters ***τ****_a_*, *T_g_*, and ***ε****_g_* as follows:

(14) $$\hat{\sum }=[\tau_{a}⊙\text{B}(T_{g})]\text{Var}({ɛ}_{g})[\text{B}(T_{g})⊙\tau_{a}]$$

where [·] denotes rearranging an *n*-vector into an *n*×*n* diagonal matrix. Thus we can re-express Equation (13) as:

(15) $$\begin{array}{ll} {\hat{\beta }}_{k} & ≅\frac{1}{{\hat{\sum }}_{kk}^{‐1}}e_{k}^{\prime }{\left[\tau_{a}\right]}^{‐1}{\left[\text{B}(T_{g})\right]}^{‐1}\text{Var}{\left({ɛ}_{g}\right)}^{‐1}{\left[\text{B}(T_{g})\right]}^{‐1}{\left[\tau_{a}\right]}^{‐1}[\tau_{a}][\text{B}(T_{p})‐{ɛ}_{g}⊙\text{B}(T_{g})]\text{A}\\ & ≅\frac{1}{{\hat{\sum }}_{kk}^{‐1}}e_{k}^{\prime }{\left[\tau_{a}\right]}^{‐1}{\left[\text{B}(T_{g})\right]}^{‐1}\text{Var}{\left({ɛ}_{g}\right)}^{‐1}([\text{B}(T_{p})][\text{B}{\left(T_{g}\right)}^{‐1}‐[{ɛ}_{g}])\text{A}\\ & =\cdots \\ & \frac{\tau_{a_{k}}\text{B}{\left(T_{g}\right)}_{k}}{\text{Var}{\left({ɛ}_{g}\right)}_{kk}^{‐1}}\sum_{i=1}^{n}\text{Var}{\left({ɛ}_{g}\right)}_{kk}^{‐1}\left(\frac{\text{B}{\left(T_{p}\right)}_{i}}{\text{B}{\left(T_{g}\right)}_{i}}‐{ɛ}_{g_{i}}\right){\text{A}}_{i} \end{array}$$

If we assume the diagonals of *Var*(***ε****_g_*)^−1^ are large compared to the off-diagonals, then Equation (15) simplifies to:

(16) $${\hat{\beta }}_{k}≅\tau_{a_{k}}(\text{B}{\left(T_{p}\right)}_{k}‐{ɛ}_{g_{k}}\text{B}{\left(T_{g}\right)}_{k})A_{k}.$$

This shows that *βˆ_k_* is proportional to *A_k_* and also depends on the temperature-emissivity contrast and atmospheric transmissivity as expected. Thus, channels where ***A*** has large absorbance features will produce detection images with strong plume pixels relative to channels where ***A*** has small absorbance features. The plume will show up in a channel because pixels that contain the plume should yield larger values of *βˆ_k_* relative to pixels that do not contain the plume (barring the effects of large deviations due to the background mean subtraction).

We note that for chemicals with sharp features in a few channels, Equation (15) says that only those channels may show the plume. For chemicals with large broad features (features spread over many channels), Equation (15) says that we may observe the plume in many of those channels. Equation (15) also shows that even if chemical ***A*** has a large absorbance in channel *k*, the atmosphere could obscure the plume signal and produce a small *βˆ_k_*. Similarly Equation (15) shows that if *ε_gk_* = *B(T_p_*)*_k_*/*B*(*T_g_*)*_k_* then *βˆ_k_* may be small. Note, this can only happen if *T_p_* < *T_g_*.

## 4. Experimental Results

We present results on synthetic hyperspectral imagery created with the Digitial Imaging and Remote Sensing Image Generator (DIRSIG) model developed at Rochester Institute of Technology (RIT). Please see [2, 7] for a detailed description of the image. We first present and discuss the synthetic image. Second we present plume detection results from applying the Matched Filter and BVD approaches.

### 4.1. Synthetic Imagery

**Figure 1.**
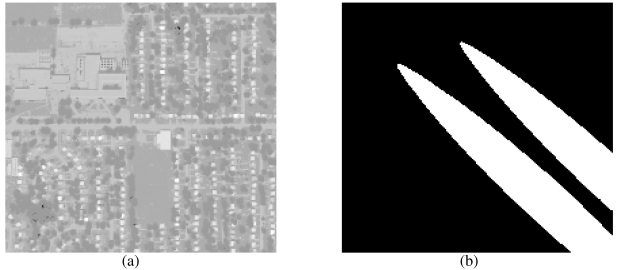
Images that show (a) a wideband picture of the synthetic DIRSIG image and (b) a mask image of the gaussian shaped NH_3_ (lower left) and Freon (upper right) plumes.

The DIRSIG image represents a highly cluttered urban scene. The image has 200 × 200 spatial pixels with 128 LWIR channels ranging from 7.5188 *μ*m to 13.605 *μ*m. This image includes two large simulated plume releases, one each for gases Freon-114 and ammonia (NH_3_). A wideband picture of the DIRSIG image and the plume mask are presented in Figure 1(a) and (b) respectively. The plume temperature and concentration path-lengths are strongest near the release point. The plume concentration path-lengths vary from approximately 70 *ppm-m* at the source to approximately 1 *ppm-m* at the lower right edge of the image. Figure 1(b) shows that the plumes cover a considerable region of the synthetic scene. We note that the two large plumes cover approximately 23% of the image.

### 4.2. Scene Whitening

It is well known that large amounts of plume information in the whitening statistics *L̄* and Σˆ will degrade the performance of matched filter detection [12]. This may be difficult to avoid in practice because eliminating plume information from the whitening statistics requires knowledge of which pixels are plume pixels. If this information is available *a priori* then detection is not necessary. To produce an informative comparison between matched filter detection and BVD, we compare them under two whitening approaches, *scene-wide* whitening and *background-only* whitening (no plume pixels). Background-only whitening is possible on our synthetic DIRSIG image because we have ground truth which allows us to remove every plume pixel from the whitening statistics. In practice, whitening usually occurs somewhere between these two extremes.

### 4.3. Matched Filter Detection Images

**Figure 2.**
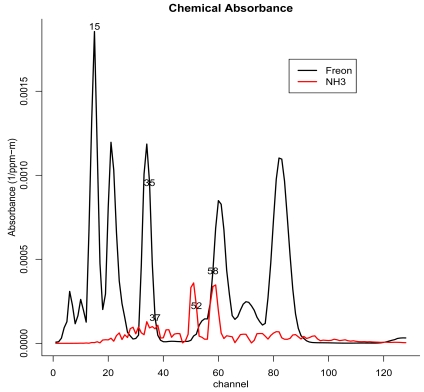
Chemical absorbance spectra for Freon-114 and NH _3_.

**Figure 3.**
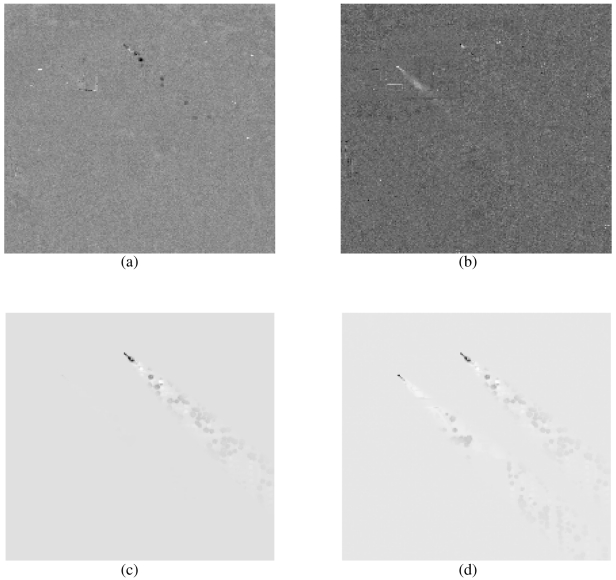
Images that show the GLS detection image for (a) Freon (scene-wide whitening), (b) NH_3_ (scene-wide whitening), (c) Freon (background-only whitening) and (d) NH_3_ (background-only whitening).

We applied the matched filter detection method as described in Section 3.1 using both Freon and NH_3_ gas absorbance spectra from the PNNL Vapor Phase Library [13]. A detection image was created for each chemical using both scene-wide and background-only whitening. These are presented in Figure 3. To aid interpretation of the results, we include the absorbance spectra for both Freon-114 and NH_3_ in Figure 2, along with notation to indicate the channels displayed later in the BVD results.

Figure 3(a) displays the detection image for Freon using scene-wide whitening. The image shows some groupings of pixels in the region of the Freon plume that exhibit visual contrast to the surrounding pixels. We consider these pixels as the matched filter evidence for the Freon plume.

Figure 3(b) displays the detection image for NH_3_ using scene-wide whitening. The image shows a grouping of pixels near the release point that have a bright contrast to the surrounding pixels. We consider these pixels as the matched filter evidence for the NH_3_ plume.

Figure 3(c) displays the detection image for Freon using background-only scene whitening. As expected, this detection image is much improved over Figure 3(a). When compared to the plume mask in 1(b) we see that even pixels at significant distance from the source stand out from the background.

Figure 3(d) displays the detection image for NH_3_ using background-only whitening. In this case we also see significant improvement over Figure 3(b) but due to the similar spectral features near channel 35 and 58 in both Freon and NH_3_, the Freon plume is just as visually prominent as the NH_3_ plume in the NH_3_ detection image. This may be an advantage in that the NH_3_ image reveals both the NH_3_ and the Freon plume, but may be misleading in the identification phase. These images will be compared to BVD detection images which we present in the next section.

### 4.4. BVD Detection Images

We applied BVD as described in Section 3.2 to the DIRSIG image. This resulted in a set of 128 images that were inspected for gray scale contrast in the region of either plume. There were multiple images that showed some contrast. We present two for each chemical plume that provide detection evidence.

**Figure 4.**
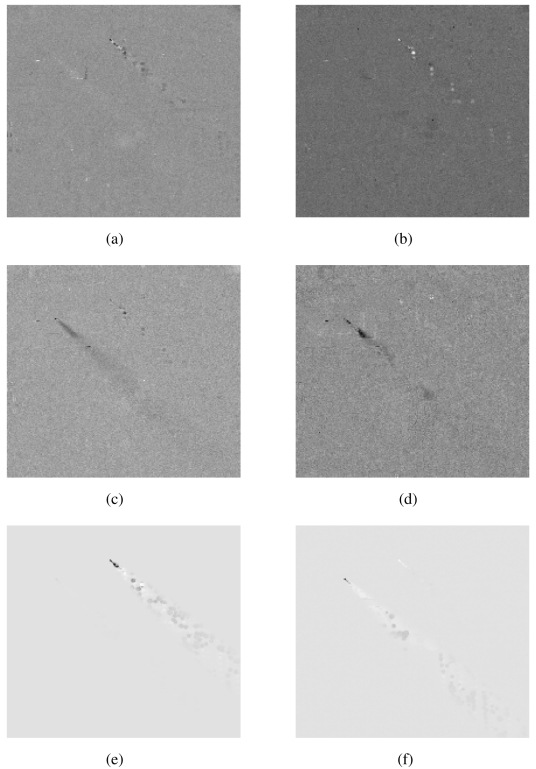
Images that show the BVD image detecting the (a) Freon plume in Channel 15 (scene-wide whitening) (b) Freon plume in Channel 35 (scene-wide whitening) (c) NH_3_ plume in Channel 37 (scene-wide whitening) (d) NH_3_ plume in Channel 58 (scene-wide whitening) (e) Freon plume in channel 35 (background-only whitening) and (f) NH_3_ plume in channel 52 (background-only whitening).

Figure 4(a),(b) shows gray scale contrast in the region of the Freon plume using scene-wide whitening. These are channels 15 and 35. The image in Figure 4(a) is similar to the matched filter Freon image in Figure 3(a). Figure 4(b) shows similar patterns but with an opposite color contrast to Figure 3(a) and Figure 4(a). Figure 4(c),(d) shows gray scale contrast in the region of the NH_3_ plume using scene-wide whitening. These are channels 37 and 58. When compared to the matched filter NH_3_ image in Figure 3(b) they show arguably better gray scale contrast in the region of the NH_3_ plume. Specifically Figure 4(d) shows a brighter collection of pixels in the region of the NH_3_ plume than does the matched filter NH_3_ image in Figure 3(b).

Figure 4 (e) shows gray scale contrast in the region of the Freon plume due to channel 35 using background-only whitening. It is a significant improvement over the scene-wide whitening results in Figure 4 (a) and (b), and it is almost identical to the background-only matched filter result for Freon in Figure 3(c). Figure 4(f) shows gray scale contrast in the region of the NH_3_ plume due to channel 52. It shows some improvement over the scene-wide result in Figure 4(c) and (d); and it is similar to the background-only matched filter result for NH_3_ in Figure 3(d), except it contains only very weak evidence of the Freon plume.

## 5. Conclusions

The purpose of this paper is to introduce the BVD method and compare it to other plume detection methods, in particular, matched filter methods based on GLS. We have shown it has potential to be part of a robust atmospheric chemical plume detection tool. How BVD fits into such a tool remains to be determined.

The BVD method does not require a chemical search library and yet it appears to detect plumes as well as the matched filter method which does depend on a chemical search library. Further study is needed in a weak plume environment. In this study, we attempted to make the comparison meaningful by looking at two cases: scene-wide and background-only whitening statistics. These cases were chosen because they represent the extreme performance expectations for matched filter detection. Scene-wide whitening is known to be the worst case for matched filter detection while background-only whitening is known to be optimal. In both cases, BVD produces visual detection images that are comparable to the matched filter detection images.

Since BVD is based on a surrogate set of spectra that span the set of all possible spectra, it is guaranteed to respond to any chemical plume without prior knowledge of the plume constituents. Detectability depends on plume strength, just as it does for every other detection method. While BVD is a plume detection method, it provides some information about the spectral content of the plume signal, which could be used as a precursor to chemical identification.

The basic BVD method presented here suggests several extensions and adaptations. The figures show selected detection images based on the *βˆ* images but initial results have shown that Bayesian methods can help sharpen detection image contrast. The BVD *βˆ* images shown here are the channels that best represent the plume but several other channels exhibit some plume evidence. Bayesian methods combined with BVD provide a way to improve contrast in the best channels by taking advantage of the weak evidence in other channels. They also tend to concentrate plume information into a smaller number of channels, making visual inspection of all channels easier for the analyst. In addition, Bayesian methods may lead to more automated detection methods, reducing the need for an analyst to inspect every detection image. We are considering other post-processing of the *βˆ* data to sharpen contrast and automate detection.

The basis set used here is the coordinate unit vectors. There are several other possible basis sets that could be used depending on the *a priori* information available. For example, suppose the user is interested in a small set of chemicals, but wants to make sure that other chemicals are not missed. The basis set could be adapted to include the small target set, and augmented with additional vectors using the Gram-Schmidt process so the whole vector space is represented. This would be a hybrid approach combining traditional matched filter detection with the BVD method presented in this paper.

In addition, we are working on other ideas related to BVD. These include using the BVD results to construct a prior distribution on the model space to improve plume identification, and using combinations of basis vectors in BVD instead of single channels.
